# Supplementary material for: Inert and seed-competent tau monomers suggest structural origins of aggregation
Source: eLife. 2018 Jul 10;7:e36584. doi: 10.7554/eLife.36584 (PMC6039173; doi:10.7554/eLife.36584)
Supplement: Figure 7—source data 2. [file elife-36584-fig7-data2.docx]

**Figure 7 source data 2. List of consensus crosslink pairs across three indendent replicates for the following conditions:** Ms 0.25 24h, Ms 0.25 3h, Ms 0.25 0h, Ms 1 24h, Ms 1 3h, Ms 1 0h, Mi 0h, Mi 3h, Mi 24h, Ms 24h, Ms 3h and Ms 0h.

**Ms_0.25_24h_consensus**

130 234

174 190

**Ms_0.25_3h_consensus**

130 140

130 150

143 150

148 274

150 163

150 174

150 225

150 254

163 174

**Ms_0.25_0h_consensus**

130 140

130 150

130 163

132 143

143 150

143 163

150 163

150 174

150 254

150 267

**Ms_1_24h_consensus**

130 140

148 174

150 163

163 174

174 190

174 281

225 370

**Ms_1_3h_consensus**

130 140

143 150

148 347

150 163

150 274

163 174

174 254

224 370

**Ms_1_0h_consensus**

130 140

140 150

140 163

143 150

143 163

150 163

150 174

150 254

150 267

150 311

**Mi_24h_consensus**

130 140

143 150

150 163

163 174

225 234

225 395

**Mi_3h_consensus**

130 140

143 150

143 163

150 163

150 174

163 174

163 180

163 190

163 224

163 225

**Mi_0h_consensus**

130 140

143 150

150 163

150 174

163 174

163 180

163 224

163 395

163 438

24 163

**Ms_24h_consensus**

**Ms_3h_consensus**

150 163

150 254

150 280

174 190

**Ms_0h_consensus**

130 140

140 225

150 163

150 174

150 274

150 311

163 174

163 225

174 225

174 240
