# Supplementary material for: Inert and seed-competent tau monomers suggest structural origins of aggregation
Source: eLife. 2018 Jul 10;7:e36584. doi: 10.7554/eLife.36584 (PMC6039173; doi:10.7554/eLife.36584)
Supplement: Transparent reporting form [file elife-36584-transrepform.docx]

***eLife’s* transparent reporting form**

We encourage authors to provide detailed information *within their submission* to facilitate the interpretation and replication of experiments. Authors can upload supporting documentation to indicate the use of appropriate reporting guidelines for health-related research (see [EQUATOR Network](http://www.equator-network.org/%20)), life science research (see the [BioSharing Information Resource](https://biosharing.org/)), or the [ARRIVE guidelines](http://www.plosbiology.org/article/info:doi/10.1371/journal.pbio.1000412) for reporting work involving animal research. Where applicable, authors should refer to any relevant reporting standards documents in this form.

If you have any questions, please consult our Journal Policies and/or contact us: [editorial@elifesciences.org](mailto:editorial@elifesciences.org).

**Sample-size estimation**

- You should state whether an appropriate sample size was computed when the study was being designed
- You should state the statistical method of sample size computation and any required assumptions
- If no explicit power analysis was used, you should describe how you decided what sample (replicate) size (number) to use

Please outline where this information can be found within the submission (e.g., sections or figure legends), or explain why this information doesn’t apply to your submission:

Overall we did not study populations either of animals or humans, and thus it seems unnecessary or not applicable to use sample size estimations for these biochemical experiments

In the case of Rosetta simulations, we arbitrarily chose 1000 as the number of simulations to test. This is relatively standard in the field. Using our computer cluster this represented 2 weeks of computer time. To substantially increase the number of simulations (e.g. by 5-10-fold) would be too expensive and impractical, and would probably not change the conclusions substantially.

**Replicates**

- You should report how often each experiment was performed
- You should include a definition of biological versus technical replication
- The data obtained should be provided and sufficient information should be provided to indicate the number of independent biological and/or technical replicates
- If you encountered any outliers, you should describe how these were handled
- Criteria for exclusion/inclusion of data should be clearly stated
- High-throughput sequence data should be uploaded before submission, with a private link for reviewers provided (these are available from both GEO and ArrayExpress)

Please outline where this information can be found within the submission (e.g., sections or figure legends), or explain why this information doesn’t apply to your submission:

**Biological Replicates:** This refers to separately generated samples, e.g. analysis of samples from different individuals, in which the input represents a distinct biological source, or analysis of fibril preparations formed independently.

**Technical Replicates:** This refers to independently analyzed samples in which each would be expected otherwise to be identical, e.g. multiple wells in which the same cell line was treated with the same sample, or when a single brain is broken up into three fractions for identical analyses.

**Outliers:** We did not exclude outliers in any case.

**Inclusion/Exclusion of data:** In no case did we include or exclude data.

**Figure 1**. Biochemical purification of M_i_ and M_s_ and seeding into cells was carried out ~50 times over 3 years. In Figure 1 the data shown is a representative dataset for M_i_ and M_s_ on SEC, seeding, microscopy and seeding following filtration. *In vitro* M_s_ and M_i_ aggregation was done once (using N=3 independent technical replicates per fraction studied). Data from the titration of M_i_ and M_s_ seeding in cells is from a single representative experiment (from at least three independent studies), using technical triplicates for each sample. Sample number is described in the methods section, and the primary data is available in Figure 1 source data 1.

**Figure 2.** CD experiments were carried out with 3 biological replicates. A single representative experiment is shown, and the data for M_i_ and M_s_ is an average of 4 technical replicates. The FCS experiment was repeated 3 times with biological replicates. A single representative scan is shown. Sample size is described in the methods section and the primary data is available in Figure 2 source data 1.

**Figure 3.** Seeding activity was measured in technical triplicates. Sample size is described in the methods and the primary data is available in Figure 3 source data 1.

**Figure 4.** Heat denaturation was done in two biological replicates, for which a representative experiment is shown. Seeding activity was performed in technical triplicates. Sample size is described in the methods, and the primary data is available in Figure 4 source data 1.

**Figure 5.** Stability of monomers and assemblies in solution was tested in a single experiment, with 32 SEC analyses. Sample size is described in the methods, and primary data is available in Figure 5 source data 1.

**Figure 6.** This single experiment is representative of at least three distinct biological replicates. Seeding activity was determined in technical triplicates (N=3). Sample size is described in the methods, and primary data is available in Figure 6 source data 1.

**Figure Supplement 6.** This experiment was performed in three biological replicates. A representative gel is shown.

**Figure 7.** Each XL-MS dataset was collected in biological triplicate across the different conditions (M_i_:Recombinant; M_s_: Fibril-derived; M_s_: Heparin-derived). The figure indicates consensus crosslinks across triplicates for each condition. Sample size is described in the methods, and primary data is available in Figure 7 source data 1, and Figure 7 source data 2.

**Figure Supplement 7.** This represents a histogram of consensus pairs across triplicates for each condition depicted in Figure 7. Sample size is described in the methods, and primary data is available in Figure 7 source data 1, and Figure 7 source data 2.

**Figure 8.** SEC was performed at least 3 times, and a single representative experiment is shown. The seeding assay was performed in technical triplicate for each fraction. Spiking of tau KO brain with fibrils or M_s_ was performed once, and the SEC was performed once, with the seeding assay performed in technical triplicate. XL-MS was performed in biological triplicate for Controls vs. AD. Sample size is described in the methods, and primary data is available in Figure 8 source data 1 and 2.

**Figure Supplement 8.** XL-MS data from different methods of homogenization was performed in 4 individual experiments on a single biological sample Sample size is described in the methods, and primary data is available primary data available in Figure 8 source data 2.

**Figure 9.** Rosetta simulations produced models from 1000 independent trajectories across four different conditions: M_i_, M_s_, Control, AD. Sample size is described in the methods, and the pymol session is available in Figure 9 source data 1.

**Figure Supplement 9.** Plot of the energetics of the ensemble of 1000 Rosetta models built for each condition. Sample size is described in the methods, and primary data is available available in Figure 9 source data 2.

**Figure 10.** Proteolysis experiments were carried out for M_i_ and M_s_ in technical triplicate for each time point. Sample size is described in the methods, and primary data is available in Figure 10 source data 1.

**Figure Supplement 10.** Summary data from Figure 10, comparing peptide abundance between Mi and Ms is represented as technical triplicates for each data point presented. Sample size is described in the methods, and primary data is available in Figure 10 source data 2.

**Statistical reporting**

- Statistical analysis methods should be described and justified
- Raw data should be presented in figures whenever informative to do so (typically when N per group is less than 10)
- For each experiment, you should identify the statistical tests used, exact values of N, definitions of center, methods of multiple test correction, and dispersion and precision measures (e.g., mean, median, SD, SEM, confidence intervals; and, for the major substantive results, a measure of effect size (e.g., Pearson's r, Cohen's d)
- Report exact p-values wherever possible alongside the summary statistics and 95% confidence intervals. These should be reported for all key questions and not only when the p-value is less than 0.05.

Please outline where this information can be found within the submission (e.g., sections or figure legends), or explain why this information doesn’t apply to your submission:

All error bars represent standard deviation throughout the manuscript. This is mentioned in the methods section.

**Figure 1.** One-way ANOVA with Bonferroni post hoc analysis was used to compare groups, and is described in the legend and methods.

**Figure 2.** Error bars represent the standard deviation of cross-correlation. This is mentioned in the legend.

**Figure 3.** Error bars represent the standard deviation of seeding activity. This is mentioned in the legend.

**Figure 4.** Error bars represent the standard deviation of seeding. This is mentioned in the legend.

**Figure 5.** N/A

**Figure 6.** Error bars represent the standard deviation of seeding. This is mentioned in the legend.

**Figure 6 Supplemental Data.** N/A

**Figure 7.** N/A

**Figure 7 Supplemental Data.** N/A

**Figure 8.** Error bars represent the standard deviation of seeding. This is mentioned in the legend. One-way ANOVA with Bonferroni post hoc analysis was used to compare groups. This is described in the methods.

**Figure 8 Supplemental Data.** N/A

**Figure 9.** N/A

**Figure 9 Supplemental Data.** N/A

**Figure 10.** Error bars represent the standard deviation of peptide abundance. This is described in the methods.

**Figure Supplement 10.** Mean and standard deviation are indicated for ratios of all peptides. This is described in the methods.

(For large datasets, or papers with a very large number of statistical tests, you may upload a single table file with tests, Ns, etc., with reference to sections in the manuscript.)

**Group allocation**

- Indicate how samples were allocated into experimental groups (in the case of clinical studies, please specify allocation to treatment method); if randomization was used, please also state if restricted randomization was applied
- Indicate if masking was used during group allocation, data collection and/or data analysis

Please outline where this information can be found within the submission (e.g., sections or figure legends), or explain why this information doesn’t apply to your submission:

**Figure 1.** Direct comparison of Mi and Ms using biochemistry and seeding was performed, described in figure legend, methods and main text.

**Figure 2.** Direct comparison of Mi and Ms using CD and FCS was performed, described in figure legend, methods and main text.

**Figure 3.** N/A

**Figure 4.** Direct comparison of M_i_ and M_s_ heat stability was performed, described in figure legend, methods and main text.

**Figure 5.** M_i_ vs M_s_ self-assembly was performed, described in figure legend, methods and main text.

**Figure 6.** Direct comparison of M_i_ and heparin induced M_s_ was performed, described in figure legend, methods and main text.

**Figure Supplement 6.** Direct comparison of Mi and heparin induced Ms was performed, described in figure legend, methods and main text.

**Figure 7.** Direct comparison of Mi and different forms of Ms using XLMS was performed, described in figure legend, methods and main text.

**Figure Supplement 7.** Direct comparison of Mi and different forms of Ms using XLMS was performed, described in figure legend, methods and main text.

**Figure 8.** Direct comparison of control and AD patient derived samples was performed, described in figure legend, methods and main text.

**Figure Supplement 8.** Comparison of AD sample across different homogenization methods was performed, described in figure legend, methods and main text.

**Figure 9.** Direct comparison of models built with constraints derived from M_i_ and M_s_ was performed, described in figure legend, methods and main text.

**Figure 10.** Direct comparison of peptides observed in M_i_ and M_s_ was performed, described in figure legend, methods and main text.

**Figure Supplement 10.** Direct comparison of M_i_ and M_s_ proteolysis profiles was performed, described in figure legend, methods and main text.

**Additional data files (“source data”)**

- We encourage you to upload relevant additional data files, such as numerical data that are represented as a graph in a figure, or as a summary table
- Where provided, these should be in the most useful format, and they can be uploaded as “Source data” files linked to a main figure or table
- Include model definition files including the full list of parameters used
- Include code used for data analysis (e.g., R, MatLab)
- Avoid stating that data files are “available upon request”

Please indicate the figures or tables for which source data files have been provided:

Figure 1 source data 1 linked to **Figure 1**

Figure 2 source data 1 linked to **Figure 2**

Figure 3 source data 1 linked to **Figure 3**

Figure 4 source data 1 linked to **Figure 4**

Figure 5 source data 1 linked to **Figure 5**

Figure 6 source data 1 linked to **Figure 6**

Figure 7 source data 1 linked to **Figure 7**

Figure 7 source data 2 linked to **Figure 7**

Figure 7 source data 1 linked to **Figure Supplement 7**

Figure 7 source data 2 linked to **Figure Supplement 7**

Figure 8 source data 1 linked to **Figure 8**

Figure 8 source data 2 linked to **Figure 8**

Figure 8 source data 1 linked to **Figure Supplement 8**

Figure 9 Parameters used for Rosetta simulations can be found in the **methods** and **Figure 7 source data 2**

Figure 9 source data 1 linked to **Figure 9**

Figure 9 source data 2 linked to **Figure Supplement 9**

Figure 10 source data 1 linked to **Figure 10** and **Figure Supplement 10**

Figure 10 source data 2 linked to **Figure Supplement 10**
